# A novel comparative evaluation of multiplex PCR panels for gastrointestinal pathogen detection: Seegene Allplex™ vs. Luminex NxTAG^®^ in clinical stool samples

**DOI:** 10.1007/s10096-025-05098-5

**Published:** 2025-03-17

**Authors:** Laura Seijas-Pereda, Ana Martín, Raquel Menchero, Carlos Rescalvo-Casas, Marcos Hernando-Gozalo, Juan Cuadros-González, Ramón Pérez-Tanoira

**Affiliations:** 1 Department of Biomedicine and Biotechnology, Faculty of Medicine, University of Alcalá, Alcalá de Henares, Spain; 2 Department of Microbiology, Príncipe de Asturias University Hospital, Alcalá de Henares, Spain; 3 Diasorin Iberia S.A, Alcobendas, Spain; 4 Department of Organic and Inorganic Chemistry, University of Alcalá, Alcalá de Henares, Spain

**Keywords:** Laboratory diagnostics, Molecular assay, Syndromic testing, Positive percentage agreement and negative percentage agreement, Healthcare impact

## Abstract

**Purpose:**

Accurate and rapid diagnosis of gastrointestinal infections is essential for effective patient management. This study compared the diagnostic performance of two multiplex PCR panels— the **Seegene Allplex™ Gastrointestinal Panels** (Seegene, Seoul, Korea) and the **Luminex NxTAG**^**®**^ **Gastrointestinal Pathogen Panel** (Luminex Corporation, Austin, Texas, a Diasorin Company)—in detecting gastrointestinal pathogens from clinical stool samples.

**Methods:**

A total of 196 stool samples, collected from patients at a Spanish hospital during 2023, were analyzed using both assays through prospective and retrospective approaches. The performance of each test was assessed based on Positive Percentage Agreement (PPA), Negative Percentage Agreement (NPA), and overall agreement. Discrepancies between methods were resolved with a third confirmatory technique when available.

**Results:**

Both assays demonstrated high overall concordance, with NPA values consistently above 95% and overall Kappa values exceeding 0.8 for most pathogens. The average PPA was greater than 89% for nearly all targets; however, lower agreement was observed for *Cryptosporidium* spp. (86.6%). Notably, discrepancies were primarily observed for certain pathogens, such as *Salmonella spp.* and *Cryptosporidium spp.*, highlighting the diagnostic challenges associated with these targets.

**Conclusion:**

To our knowledge, this is the first study to compare the novel Luminex NxTAG^®^ panels from Diasorin with the Seegene Allplex™. Both multiplex assays provide rapid and reliable detection of gastrointestinal pathogens, making them valuable tools in clinical diagnostics. Future research should focus on improving detection accuracy for challenging pathogens and expanding target panels to further enhance patient management and reduce disease burden.

## Introduction

Diarrhoeal disease is a leading cause of child mortality and morbidity globally, as reported by the World Health Organization (WHO) [1]. It also affects other age groups, contributing significantly to the global disease burden — a problem that remains persistent over time [2]. As a common gastrointestinal illness, it is a frequent reason for medical consultations, even in higher-income countries, placing substantial demands on healthcare resources [3]. Infections by gastrointestinal microorganisms are among the primary causes of diarrhoea. A wide range of bacterial, viral, and parasitic pathogens can trigger symptoms in affected patients, making the effective management and prevention of these infections critical for controlling gastrointestinal diseases worldwide [4]. Notably, the primary bacterial pathogens implicated in infectious diarrhoea include *Campylobacter spp.*, diarrhoeagenic *Escherichia coli*, and *Salmonella spp.*. Other key pathogens responsible for gastrointestinal infections include *Clostridiodes difficile*, *Cryptosporidium spp.*, Rotavirus, Norovirus, and Adenovirus [5–7].

Therefore, identifying the correct causative agent becomes imperative and often requires a combination of various diagnostic tools. Traditionally, gastrointestinal bacteria and viruses were detected through stool sample cultivation on specific and sometimes challenging media, which typically took a minimum of 2–3 days and lacked high specificity. Additional morphological, biochemical, and serological testing was then required to identify and confirm the suspected culture isolates. For parasitic microorganisms, light microscopy with specific stains was commonly used; however, this method has low sensitivity and relies heavily on the expertise of the technologist. Moreover, the accuracy of these methods could be significantly compromised if the specimen was not freshly collected, if the patient had used antibiotics, or due to improper sample collection [4, 8, 9].

In this context, molecular methods have emerged as a promising approach for the diagnosis of gastrointestinal infections. Polymerase Chain Reaction (PCR) assays enable the detection of genetic material from gastrointestinal pathogens with significantly greater sensitivity and specificity than traditional culture-based methods. Their ease of use allows for increased patient testing and facilitates the identification of organisms for which no practical laboratory tests previously existed. Additionally, they overcome key limitations of conventional methods, such as specimen degradation and prolonged turnaround times [4, 10].

Numerous PCR-based assays have been developed to target specific genetic regions of enteric bacterial pathogens. These methods have been successfully implemented for several years, particularly in high-income countries [11]. Among the most widely used commercial kits are the BioFire FilmArray Gastrointestinal (GI) panel, the Seegene Allplex™ Gastrointestinal Assay, and the Luminex Gastrointestinal Pathogen Panels (GPP), including xTAG^®^ panels [11, 12]. These multiplex PCR tests allow for the simultaneous detection of multiple gastrointestinal microorganisms, offering several advantages, including faster turnaround times, comprehensive pathogen identification, and improved diagnostic accuracy. As a result, they have become valuable tools in clinical microbiology and patient management [13–15].

The selection of an appropriate diagnostic technique becomes increasingly complex as more options emerge. The chosen test is crucial in ensuring diagnostic accuracy and directly influences patient outcomes as well as laboratory management. In this study, we aim to compare the Positive Percentage Agreement (PPA) and Negative Percentage Agreement (NPA) of two multiplex molecular tests: the **Seegene Allplex™ Gastrointestinal Panels** (Seegene, Seoul, Korea) and the **Luminex NxTAG**^**®**^ **Gastrointestinal Pathogen Panel** (Luminex Corporation, Austin, Texas, a Diasorin Company). The results of this comparison will provide insights into the performance and reliability of these widely used diagnostic tools, potentially guiding clinical decision-making and improving gastrointestinal infection management.

## Materials and methods

This study compared two molecular diagnostic solutions: the Luminex NxTAG^®^ Gastrointestinal Pathogen Panel (GPP) from Diasorin and the Allplex™ multiplex gastrointestinal panels from Seegene. The study was conducted at the microbiology laboratory of Príncipe de Asturias University Hospital in Madrid, Spain, during the third trimester of 2023. A total of 196 human fecal samples submitted for clinical diagnostic purposes were included. Upon arrival, all stool samples were preserved in Cary-Blair medium and subsequently underwent genetic material extraction using the HAMILTON STARlet extraction system (Hamilton Company, USA).

We divided the study into two groups, analyzing all 196 samples using both diagnostic platforms. In the first phase, a subset of samples was selected *retrospectively* based on prior results obtained with the Seegene Allplex™ PCR. This multiplex assay comprises four panels designed for the comprehensive detection of various gastrointestinal microorganisms:

1. **GI-Bacteria (I) Assay**: *Aeromonas* spp., *Campylobacter* spp., *Clostridioides difficile* toxin B, *Salmonella* spp., Enteroinvasive *Escherichia coli / Shigella* spp., *Vibrio* spp., and *Yersinia enterocolitica*.
2. **GI-Bacteria (II) Assay**: Enteroaggregative *E. coli* (EAEC), Enteropathogenic *E. coli* (EPEC), *E. coli* O157, Enterotoxigenic *E. coli* (ETEC), Hypervirulent *C. difficile*, Enterohemorrhagic *E. coli* (EHEC).
3. **GI-Parasite**: *Blastocystis hominis*,* Giardia lamblia*,* Dientamoeba fragilis*,* Entamoeba histolytica*,* Cyclospora cayetanensis*, and *Cryptosporidium* spp.
4. **GI-Virus**: Astrovirus, Sapovirus, Rotavirus A, Norovirus GI-GII, and Adenovirus F.

Each sample required four tubes to complete the full panel detection with Seegene Allplex™. Based on these results, 60 samples, including both positive and negative specimens, were selected for the retrospective part. The original specimens were then reprocessed following the pre-treatment procedure specified in the Luminex NxTAG^®^ GPP package insert. After pre-treatment, genetic material was re-extracted using the HAMILTON STARlet automated system. The extracted material was subsequently analyzed using the Luminex NxTAG^®^ Gastrointestinal Pathogen Panel (Luminex Molecular Diagnostics, Inc., Canada). Unlike the Seegene assay, this multiplex PCR panel requires only a single tube per sample for comprehensive pathogen detection and provides broad coverage of gastrointestinal microorganisms, including:

1. **Bacteria**: *Campylobacter* spp., *C. difficile* (toxin A/B), Enterotoxigenic *E. coli* (ETEC), Shiga toxin-producing *E. coli* (*stx1*,* stx2*) (STEC*), *Shigella* spp./Enteroinvasive *E. coli*, *Salmonella* spp., *Vibrio cholerae*, and *Yersinia enterocolitica*. *The STEC group includes all the EHEC and *E. coli* O157.
2. **Parasites**: *Cryptosporidium* spp., *E. histolytica*, and *G. lamblia*.
3. **Virus**: Adenovirus F40/41, Astrovirus, Norovirus GI/GII, Rotavirus A, Sapovirus GI/GII/GIV/GV.

This preselection facilitated a focused comparison of PCR results and performance while also enabling the evaluation of the significance of the pre-treatment step included in the NxTAG GPP Luminex protocol.

The subsequent stool samples included in the study were processed using both techniques in parallel in a *prospective* manner. In total, 136 samples were included in this phase of the study, comprising both negative samples and those positive for one or more gastrointestinal microorganisms. All samples underwent the pre-treatment procedure specified in the Luminex NxTAG^®^ GPP package insert, followed by genetic material extraction using the HAMILTON STARlet automated system. The resulting eluate from each sample was then analyzed simultaneously using both PCR assays, allowing for a direct comparison of their results. This approach ensured a consistent starting material for both assays, minimizing variability in sample preparation and enabling a robust evaluation of the two PCR platforms.

Samples were classified as negative only when both techniques consistently identified them as such, although it is important to note that the number of targets detected by each method differs. Comparisons were made exclusively for microorganisms detectable by both techniques. In cases of discrepancies between the two methods, and when sufficient sample material or DNA was available, additional tests were performed to verify the accuracy of the results. Depending on the microorganism in question and the availability of confirmatory tests, this third analysis included microbial culture with plate-based identification, specific PCR assays provided by the Spanish National Center for Microbiology, or the VIASURE Real-Time PCR Detection Kit (Certest Biotec S.L., Zaragoza, Spain), targeting the microorganism of interest. Samples initially classified as “invalid” were retested and confirmed before being definitively categorized.

Statistically, since no absolute gold standard was available for comparison, Positive Percentage Agreement (PPA) and Negative Percentage Agreement (NPA) were used to assess concordance between the two diagnostic methods.

## Results

In the *retrospective* part of the study, with 60 samples included, we found the following gastrointestinal microorganisms: 10 *Campylobacter* spp., 10 *C. difficile* toxin B, 6 *Salmonella* spp., 3 Enteroinvasive *E. coli / Shigella* spp., 1 *Y. enterocolitica*, 3 ETEC, 3 STEC, 5 *Cryptosporidium* spp., 2 Rotavirus, 3 Norovirus, and 2 Adenovirus. We identified 13 negative samples using the Seegene assay and 23 negative samples using the Luminex assay. Notably, 1 positive sample according to Seegene, was classified as “Invalid” by Luminex NxTAG. The discrepancies observed were as follows: 1 *Campylobacter* spp. detected only by Seegene, and 2 *C. difficile* toxin B, 3 *Salmonella* spp., and 2 *Cryptosporidium* spp. detected only by Luminex NxTAG. Due to insufficient sample volume, we were unable to resolve these discrepancies, so all microorganisms detected by either technique were included in our analysis. (A detailed summary of the results obtained from both prospective and the retrospective part is presented in Table 1).

**Table 1:**
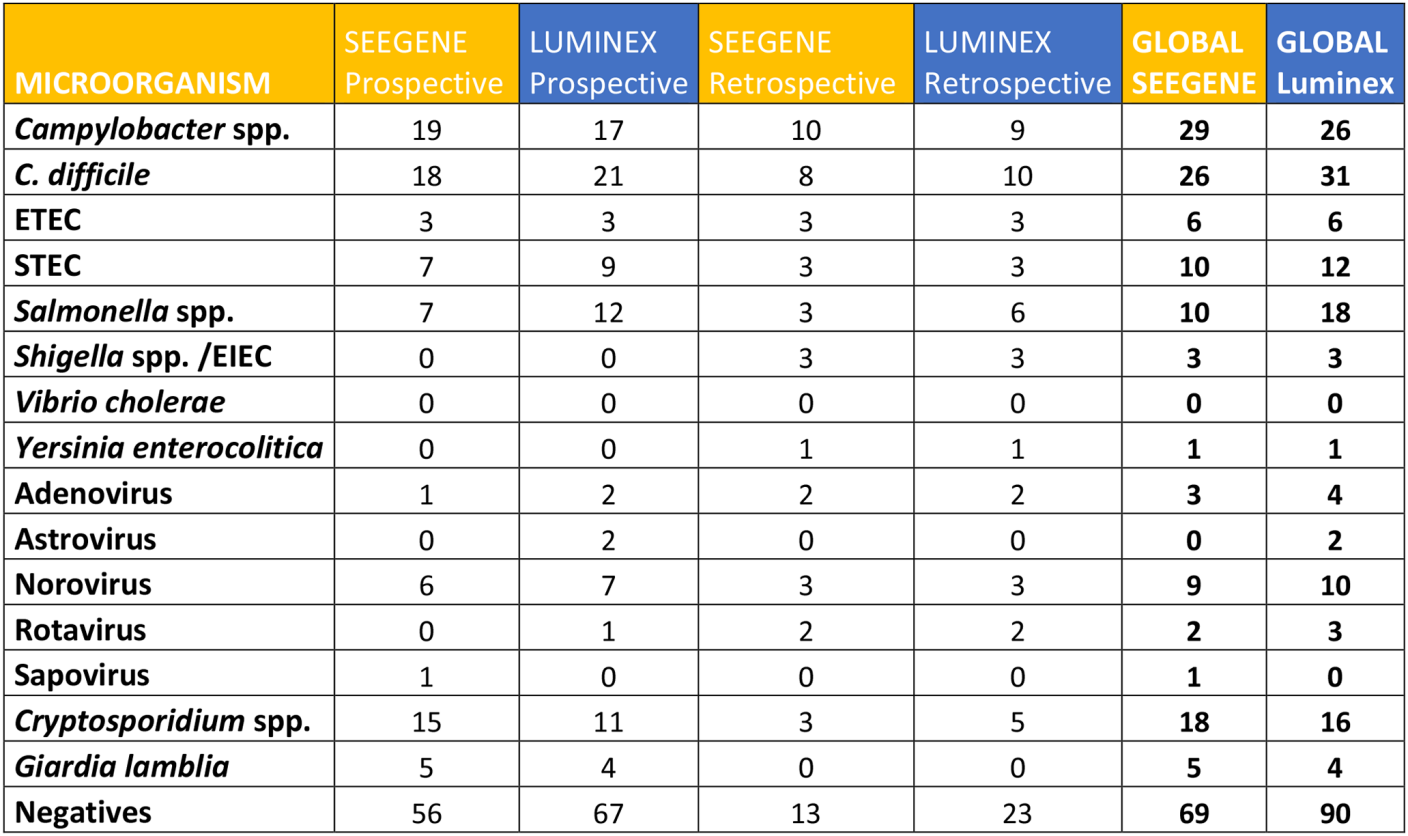
Total number of microorganisms detected by Seegene Allplex™ and Luminex NxTAG^®^ in retrospective, prospective, and overall analyses. Only microorganisms detectable by both techniques are included, while those not identified by either method are excluded. The total counts for each technique are highlighted in bold. Negative samples refer to those classified as such by each respective method

Statistically, using Seegene as the reference technique, all PPAs between the two techniques were 100%, except for *Campylobacter* spp., which had a PPA of 90%. Regarding NPAs, all were 100% except for *C. difficile* at 95.9%, *Salmonella* spp. at 94.4%, and *Cryptosporidium* spp. at 96.3%. The Kappa values for the comparisons indicated nearly perfect concordance (Kappa 0.80–1.00) for all pathogens, with the exception of *Salmonella* spp. (Kappa = 0.64) and *Cryptosporidium* spp. (Kappa = 0.73). Refer to Table 2 for detailed results on PPAs, NPAs, and Kappa values per pathogen.

**Table 2:**
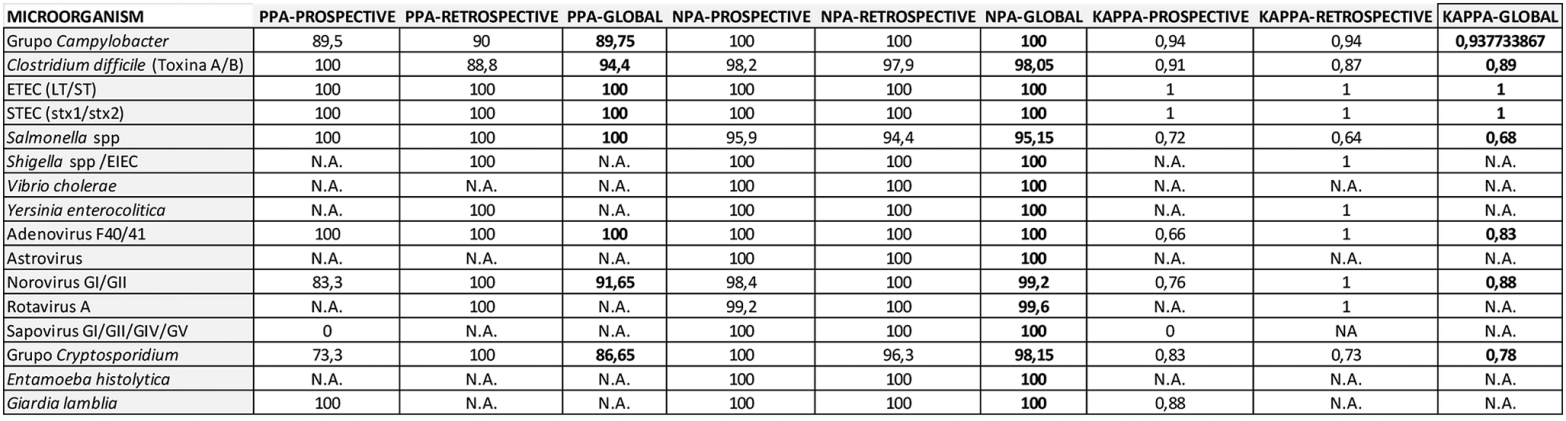
Positive percentage agreement (PPA), Negative percentage agreement (NPA), and Kappa values comparing Seegene Allplex™ and Luminex NxTAG^®^ for detected microorganisms. Data include prospective, retrospective, and overall (global) analyses, with global values in bold. Comparisons not feasible due to limited sample sizes are indicated as N.A

In the *prospective* part of the study combining both techniques in 136 samples, we identified the following microorganisms: 21 *C. difficile* toxin B, 19 *Campylobacter* spp., 12 *Salmonella* spp., 15 *Cryptosporidium* spp., 8 Norovirus, 7 STEC, 5 *G. lamblia*, 3 ETEC, 2 Adenovirus, 1 Rotavirus and 1 Sapovirus. We found 56 negative samples using the Seegene assay and 67 negative samples using the Luminex assay (Table 1).

In this phase of the study, discrepancies between the Seegene Allplex and Luminex NxTAG assays were observed in a small number of cases, with 15 out of 19 discrepancies resolved, allowing differentiation between correctly and incorrectly identified microorganisms. Specifically, *Salmonella spp.* was detected in five cases by Luminex NxTAG but not by Seegene Allplex or the third confirmatory method. Similarly, *Cryptosporidium spp.* was identified by Seegene Allplex in two cases, with only one confirmed by the third technique. *C. difficile* was detected in three cases by Luminex NxTAG, with only one confirmed by the third method. *Campylobacter spp.* was identified in two cases by Seegene Allplex, both confirmed by the third technique. For *Giardia lamblia*, *Sapovirus*, and *Adenovirus*, only one case each was identified by a single technique, with partial confirmation by the third method. Notably, three samples classified as negative by Seegene Allplex were categorized as “Invalid” by Luminex NxTAG. Statistically, PPA values were 100% for most targets, with exceptions for *Campylobacter spp.* (89.5%), Norovirus (83.3%), *Cryptosporidium spp.* (73.3%), and Sapovirus (0%, *n* = 1). NPA values remained at 100% for all microorganisms, except for *C. difficile* (98.2%), *Salmonella spp.* (95.9%), Norovirus (98.4%), and Rotavirus A (99.2%). Kappa values indicated nearly perfect concordance (Kappa > 0.80) for all pathogens except for *Salmonella spp.* (Kappa = 0.64), *Cryptosporidium spp.* (Kappa = 0.73), Adenovirus (Kappa = 0.66), and Norovirus (Kappa = 0.76) (Table 2).

Additionally, differences were observed in the detection of mixed infections between the two techniques. Using Seegene Allplex™, the overall prevalence of mixed infections was 12.76% (25/196 samples), compared to 14.29% (28/196 samples) with Luminex NxTAG®. Moreover, no single microorganism consistently dominated the coinfections; instead, a variety of pathogen combinations were observed across the samples.

## Discussion

In this study, we evaluated and compared the performance of two multiplex molecular tests for the detection of gastrointestinal microorganisms in stool samples: the **Seegene Allplex™ Gastrointestinal Panels** (Seegene, Seoul, Korea) and the **Luminex NxTAG**^**®**^ **Gastrointestinal Pathogen Panel** (Luminex Corporation, Austin, Texas, a Diasorin Company). Both panels demonstrated significant utility in clinical diagnostics, enabling the rapid and accurate identification or exclusion of a wide range of gastrointestinal pathogens, including those involved in coinfections. The use of these multiplex panels facilitated a more precise and expedited assessment of patients’ clinical symptoms, which can ultimately reduce the disease burden, minimize the risk of inappropriate therapies, and optimize the use of time and resources within healthcare systems [3, 16, 17].

In terms of concordance, our results demonstrate excellent agreement between the two techniques for most microorganisms, with Kappa values exceeding 0.8. However, lower Kappa values were observed for *Salmonella* spp., *Cryptosporidium* spp., and certain gastrointestinal viruses. Notably, differences in agreement were observed between the retrospective and prospective parts of the study for viral pathogens. In the retrospective phase, excellent agreement (Kappa = 1) was noted for Adenovirus and Norovirus, while in the prospective phase, only good concordance was achieved (Kappa < 0.8) for the same viruses. These discrepancies may be attributed to the limited sample size in each phase of the study. This finding suggests that while these multiplex panels generally provide reliable results, caution should be exercised when interpreting findings for these specific pathogens, regardless of the panel used.

Overall, both methods demonstrated high percentage agreement rates for both positive and negative results across the microorganisms studied, consistent with previous research [18]. However, discrepancies were noted in the detection of specific pathogens based on Positive Percentage Agreement (PPA) values. For instance, in the case of *Campylobacter* spp., two samples that tested positive by Seegene Allplex were confirmed by a third molecular method but were negative by Luminex NxTAG. For Norovirus, one sample positive by Seegene Allplex was negative by Luminex NxTAG; however, this sample could not be further evaluated due to insufficient volume. Similarly, for *Cryptosporidium* spp., four samples positive by Seegene Allplex were negative by Luminex NxTAG, with two of these discrepancies remaining unresolved because of insufficient sample volume.

When evaluating the exclusion of microorganisms (NPA values), minor disagreements were observed. For example, five out of 196 samples were positive for *C. difficile* by Luminex NxTAG but negative by Seegene Allplex; two of these discrepancies could not be analyzed further. In the case of *Salmonella* spp., Luminex NxTAG detected eight false-positive samples that were negative by both other methods. Additionally, two samples were positive for *Cryptosporidium* spp. by Luminex NxTAG but negative by Seegene Allplex, and two samples were positive for Norovirus by Luminex NxTAG but negative by Seegene Allplex; in both cases, insufficient sample volume precluded further analysis. For Rotavirus, the sole positive sample was detected only by Luminex NxTAG and could not be confirmed by a third method due to limited sample volume.

These discrepancies, consistent with other studies [10], underscore the challenges in accurately detecting the genetic material of these pathogens. Factors contributing to these differences may include variations in the specific genomic regions targeted and differences in primer design between the two techniques. Such genomic regions can exhibit polymorphisms, which are particularly common in certain microorganisms like *Cryptosporidium* spp., ultimately affecting the performance of different diagnostic platforms [19].

Previous investigations have reported high sensitivity and specificity rates for the Seegene Allplex panels, with some studies noting up to 98.3% sensitivity compared to other molecular methods and traditional microbiological techniques [10, 20, 21] Similarly, the original Luminex xTAG^®^ GPP has demonstrated robust diagnostic accuracy, with comparable performance metrics [22, 23]. However, this study is the first to directly compare the Seegene Allplex panel with the novel Luminex NxTag technology. Notably, the pathogens with lower concordance in our study—such as those mentioned—are consistent with those identified as problematic when comparing Seegene and Luminex with other molecular techniques. This suggests that their DNA may present specific challenges for accurate detection. Future investigations should focus on addressing these challenges to further improve the diagnostic precision of these multiplex panels.

Regarding coinfections, both techniques identified similar percentages: 12.76% with Seegene and 14.29% with Luminex NxTAG. These values are consistent with, or even exceed, those reported in the literature for multiplex molecular panels [24]. This underscores the importance of simultaneously detecting multiple microorganisms in each sample, as targeted assays might overlook certain infections.

In summary, the Seegene Allplex is a multiplex RT-PCR assay composed of four independent panels (Bacteria I, Bacteria II, Viruses, and Parasites) that can be selected based on the patient’s clinical presentation. The throughput of the Seegene test depends on the number of panels used; when all four panels are employed, it can detect up to 24 microorganisms. In contrast, the Luminex NxTAG GPP is a multiplex PCR assay designed to simultaneously detect 16 different targets (bacteria, viruses, and parasites) in a single tube, thereby enabling the processing of up to 96 samples per PCR run.

These findings have significant implications for clinical practice. While both multiplex panels provide rapid and reliable results for most microorganisms, clinicians should be aware of their limitations. Selecting the most appropriate diagnostic tool—especially in complex cases or when dealing with pathogens that are more challenging to detect—is essential to ensure accurate diagnosis and effective treatment.

The limitations of our study include its single-center design, the relatively small sample size, the lack of comprehensive clinical and epidemiological data, and the inability to resolve discrepancies in the retrospective part of the study. These factors may affect the generalizability of our findings. Future multicenter studies with larger sample sizes and more detailed clinical data are needed to validate our results and address these limitations.

## Conclusion

In summary, our comparison of the Seegene Allplex™ Gastrointestinal Panels and the Luminex NxTAG^®^ Gastrointestinal Pathogen Panel revealed high concordance rates for most gastrointestinal pathogens, confirming the utility of these multiplex molecular tests in clinical diagnostics. Nevertheless, challenges remain in molecular diagnostics, as evidenced by discrepancies in the detection of certain pathogens. Such discrepancies underscore the need for cautious interpretation of results, particularly in cases where specific pathogens are clinically suspected.

Future research should aim to refine these molecular techniques to overcome the identified challenges, enhance detection accuracy for problematic pathogens, and ultimately improve patient outcomes. Additionally, expanding investigations to include a broader range of pathogens and diverse clinical settings will further validate the robustness and applicability of these diagnostic panels.

Overall, these multiplex assays represent a valuable tool for the simultaneous diagnosis of multiple infections, as they provide semi-quantitative data that supports more accurate result interpretation and comprehensive validation across a wide array of pathogens.

## Data Availability

No datasets were generated or analysed during the current study.
